# (4*Z*)-4-[(2*E*)-1-Hy­droxy-3-(3-nitro­phen­yl)prop-2-en-1-yl­idene]-3-methyl-1-(4-methyl­phen­yl)-1*H*-pyrazol-5(4*H*)-one

**DOI:** 10.1107/S1600536812025238

**Published:** 2012-06-13

**Authors:** Faryal Chaudhry, M. Nawaz Tahir, Misbahul Ain Khan, Abdul Qayyum Ather, Nadia Asif

**Affiliations:** a Institute of Chemistry, University of the Punjab, Lahore, Pakistan; b University of Sargodha, Department of Physics, Sargodha, Pakistan; c Department of Chemistry, Islamia University, Bahawalpur, Pakistan; d Applied Chemistry Research Center, PCSIR Laboratories Complex, Lahore 54600, Pakistan

## Abstract

In the title compound, C_20_H_17_N_3_O_4_, the dihedral angles between the heterocyclic ring and the toluene and nitro­benzene rings are 4.21 (15) and 11.43 (14)°, respectively. The whole mol­ecule is close to planar (r.m.s. deviation for the 27 non-H atoms = 0.171 Å). Two *S*(6) rings are formed due to intra­molecular C—H⋯O and O—H⋯O hydrogen bonds. In the crystal, inversion dimers linked by pairs of C—H⋯O bonds generate *R*_2_^2^(10) loops and further C—H⋯O bonds link the dimers along the *b*-axis direction. There exist π–π inter­actions between the heterocyclic rings at a centroid–centroid distance of 3.7126 (10) Å and between the centroids of the benzene rings at a distance of 3.8710 (16) Å.

## Related literature

For background and a related structure, see: Mukhtar *et al.* (2010 ▶). For other related structures, see: Udaya Lakshmi *et al.* (2005 ▶); Jadeja & Shah (2007 ▶).
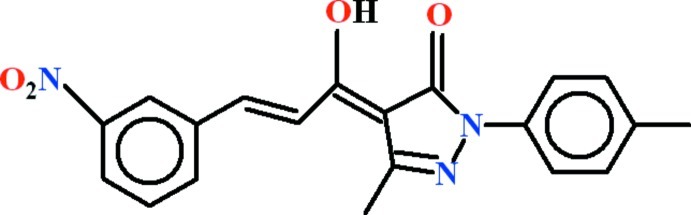


## Experimental

### 

#### Crystal data

C_20_H_17_N_3_O_4_
*M* *_r_* = 363.37
Monoclinic, 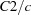

*a* = 19.8712 (16) Å
*b* = 12.1917 (10) Å
*c* = 16.733 (2) Å
β = 121.188 (4)°
*V* = 3467.9 (6) Å^3^
*Z* = 8
Mo *K*α radiation
μ = 0.10 mm^−1^
*T* = 296 K
0.35 × 0.18 × 0.17 mm

#### Data collection

Bruker Kappa APEXII CCD diffractometer
Absorption correction: multi-scan (*SADABS*; Bruker, 2005 ▶) *T*_min_ = 0.972, *T*_max_ = 0.983
12231 measured reflections
3328 independent reflections
1823 reflections with *I* > 2σ(*I*)
*R*_int_ = 0.039

#### Refinement

*R*[*F* ^2^ > 2σ(*F* ^2^)] = 0.052
*wR*(*F* ^2^) = 0.143
*S* = 1.02
3328 reflections
247 parameters
H-atom parameters constrained
Δρ_max_ = 0.16 e Å^−3^
Δρ_min_ = −0.22 e Å^−3^

### 

Data collection: *APEX2* (Bruker, 2009 ▶); cell refinement: *SAINT* (Bruker, 2009 ▶); data reduction: *SAINT*; program(s) used to solve structure: *SHELXS97* (Sheldrick, 2008 ▶); program(s) used to refine structure: *SHELXL97* (Sheldrick, 2008 ▶); molecular graphics: *ORTEP-3 for Windows* (Farrugia, 1997 ▶) and *PLATON* (Spek, 2009 ▶); software used to prepare material for publication: *WinGX* (Farrugia, 1999 ▶) and *PLATON*.

## Supplementary Material

Crystal structure: contains datablock(s) global, I. DOI: 10.1107/S1600536812025238/hb6835sup1.cif

Structure factors: contains datablock(s) I. DOI: 10.1107/S1600536812025238/hb6835Isup2.hkl

Supplementary material file. DOI: 10.1107/S1600536812025238/hb6835Isup3.cml

Additional supplementary materials:  crystallographic information; 3D view; checkCIF report

## Figures and Tables

**Table 1:** Hydrogen-bond geometry (Å, °)

*D*—H⋯*A*	*D*—H	H⋯*A*	*D*⋯*A*	*D*—H⋯*A*
O2—H2*A*⋯O1	0.82	1.82	2.571 (3)	152
C6—H6⋯O1	0.93	2.33	2.958 (3)	125
C18—H18⋯O4^i^	0.93	2.57	3.496 (3)	171
C20—H20⋯O3^ii^	0.93	2.43	3.172 (3)	136

Symmetry codes: (i) 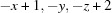; (ii) 
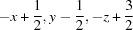
.

**Fig. 1.**
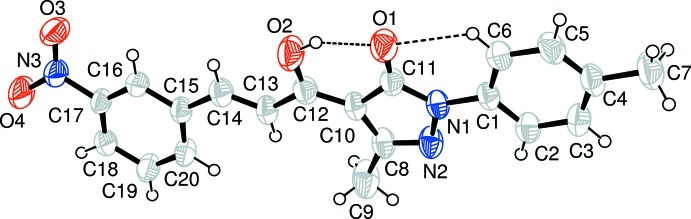
View of the title compound with displacement ellipsoids drawn at the 50% probability level. The dotted lines represents the intramolecular H-bondings.

**Fig. 2.**
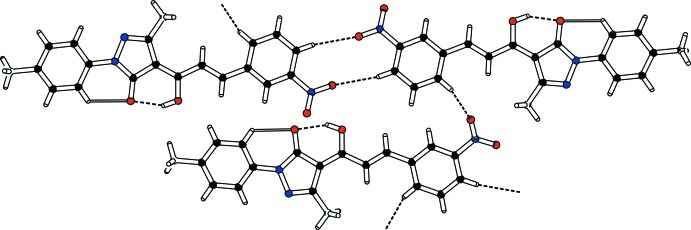
The partial packing, which shows that molecules form dimers.

## supplementary crystallographic information

### Comment

We have reported the synthesis and crystal structure of (II) *i.e.*, Ethyl 2-benzamido-4,5,6,7-tetrahydro-1-benzothiophene-3-carboxylate (Mukhtar *et al.*, 2010). The title compound (I, Fig. 1) is being reported here in continuation to synthesize various thiophene derivatives.

The crystal structure of 3-nitrocinnamic acid (Udaya Lakshmi *et al.*, 2005) and 5-methyl-4-(phenyl(phenylamino)methylene)-2-*p*-tolyl-2,4-dihydropyrazol-3-one (Jadeja & Shah, 2007) have been published which contain the fragments of the title compound.

In (I), the toluene group A (C1—C7) and the part of 5-methyl-2,4-dihydro- 3*H*-pyrazol-3-one B (C8—C11/N1/N2/O1) are planar with r.m.s. deviation of 0.0052 and 0.0171 Å, respectively. The dihedral angle between A/B is 4.42 (10)°. The part of 3-nitrocinnamic acid C (C12—C20/N3/O2/O3/O4) has r. m. s. deviation of 0.1229 Å from the plane in which O3 atom deviate to 0.2632 (17) Å. The dihedral angle between A/C abd B/C is 7.93 (6) and 12.26 (7)°, respectively. In the title compound two *S*(6) ring motifs are formed due to intramolecular H-bondings of C—H···O and O—H···O types (Table 1, Fig. 1). The molecules are dimerized from nitrobenzene due to C—H···O type of H-bondings and form *R*_2_^2^(10) ring motifs (Table 1, Fig. 2). The dimers are again interlinked from nitrobenzene due to C—H···O type of H-bondings. There exist π···π interaction between *Cg*1···*Cg*1^i^ [i = - *x*, *y*, 1/2 - *z*] at a distance of 3.7126 (10) Å, where *Cg*1 is the centroid of heterocyclic five membered ring (N1/N2/C8/C10/C11). Similarly, there also exist π···π interaction between *Cg*2···*Cg*3^ii^ [ii = - *x*, - *y*, 1 - *z*] and *Cg*3···*Cg*2^ii^ at a distance of 3.8710 (16) Å, where *Cg*2 and *Cg*3 are the centroids of benzene rings (C1—C6) and (C15—C20).

### Experimental

1-(5-Hydroxy-3-methyl-1-*p*-tolyl-1*H*-pyrazol-4-yl)ethanone (1 g, 4.34 mmol), *m*-nitrobenzaldehyde (0.65 g; 4.34 mmol), with one drop of piperidine in ethanol (30 ml) was heated on boiling water bath for half an hour and crude product was obtained on cooling. The product was recrystallized by ethanol to get orange needles of the title compound.

### Refinement

The H-atoms were positioned geometrically (O—H = 0.82, C–H = 0.93–0.96 Å) and refined as riding with *U*_iso_(H) = *xU*_eq_(C, O), where *x* = 1.5 for methyl and *x* = 1.2 for all other H-atoms.

### Crystal data

**Table:**

C_20_H_17_N_3_O_4_	*F*(000) = 1520
*M**_r_* = 363.37	*D*_x_ = 1.390 Mg m^−^^3^
Monoclinic, *C*2/*c*	Mo *K*α radiation, λ = 0.71073 Å
Hall symbol: -C 2yc	Cell parameters from 1823 reflections
*a* = 19.8712 (16) Å	θ = 2.1–26.0°
*b* = 12.1917 (10) Å	µ = 0.10 mm^−^^1^
*c* = 16.733 (2) Å	*T* = 296 K
β = 121.188 (4)°	Rod, orange
*V* = 3467.9 (6) Å^3^	0.35 × 0.18 × 0.17 mm
*Z* = 8	

### Data collection

**Table:**

Bruker Kappa APEXII CCD diffractometer	3328 independent reflections
Radiation source: fine-focus sealed tube	1823 reflections with *I* > 2σ(*I*)
Graphite monochromator	*R*_int_ = 0.039
Detector resolution: 8.00 pixels mm^-1^	θ_max_ = 26.0°, θ_min_ = 2.1°
ω scans	*h* = −22→24
Absorption correction: multi-scan (*SADABS*; Bruker, 2005)	*k* = −15→15
*T*_min_ = 0.972, *T*_max_ = 0.983	*l* = −20→13
12231 measured reflections	

### Refinement

**Table:**

Refinement on *F*^2^	Primary atom site location: structure-invariant direct methods
Least-squares matrix: full	Secondary atom site location: difference Fourier map
*R*[*F*^2^ > 2σ(*F*^2^)] = 0.052	Hydrogen site location: inferred from neighbouring sites
*wR*(*F*^2^) = 0.143	H-atom parameters constrained
*S* = 1.02	*w* = 1/[σ^2^(*F*_o_^2^) + (0.0688*P*)^2^] where *P* = (*F*_o_^2^ + 2*F*_c_^2^)/3
3328 reflections	(Δ/σ)_max_ < 0.001
247 parameters	Δρ_max_ = 0.16 e Å^−^^3^
0 restraints	Δρ_min_ = −0.22 e Å^−^^3^

### Special details

**Table:**

Geometry. Bond distances, angles *etc*. have been calculated using the rounded fractional coordinates. All su's are estimated from the variances of the (full) variance-covariance matrix. The cell e.s.d.'s are taken into account in the estimation of distances, angles and torsion angles
Refinement. Refinement of *F*^2^ against ALL reflections. The weighted *R*-factor *wR* and goodness of fit *S* are based on *F*^2^, conventional *R*-factors *R* are based on *F*, with *F* set to zero for negative *F*^2^. The threshold expression of *F*^2^ > σ(*F*^2^) is used only for calculating *R*-factors(gt) *etc*. and is not relevant to the choice of reflections for refinement. *R*-factors based on *F*^2^ are statistically about twice as large as those based on *F*, and *R*- factors based on ALL data will be even larger.

### Fractional atomic coordinates and isotropic or equivalent isotropic displacement parameters (Å^2^)

**Table:**

	*x*	*y*	*z*	*U*_iso_*/*U*_eq_	
O1	−0.08035 (9)	0.17748 (13)	0.26833 (13)	0.0730 (6)	
O2	0.04784 (9)	0.17674 (14)	0.42872 (13)	0.0821 (7)	
O3	0.36766 (10)	0.22769 (14)	0.89614 (12)	0.0776 (7)	
O4	0.45928 (10)	0.10845 (15)	0.95844 (14)	0.0945 (8)	
N1	−0.12499 (10)	−0.00138 (14)	0.21735 (13)	0.0471 (6)	
N2	−0.09808 (10)	−0.10774 (14)	0.24985 (14)	0.0569 (7)	
N3	0.39439 (11)	0.13844 (17)	0.89657 (14)	0.0581 (8)	
C1	−0.19601 (12)	0.01116 (18)	0.12989 (15)	0.0455 (8)	
C2	−0.23410 (13)	−0.0800 (2)	0.07708 (16)	0.0553 (8)	
C3	−0.30350 (13)	−0.0687 (2)	−0.00721 (17)	0.0605 (9)	
C4	−0.33702 (12)	0.0320 (2)	−0.04231 (17)	0.0580 (9)	
C5	−0.29776 (13)	0.1220 (2)	0.01114 (18)	0.0625 (9)	
C6	−0.22799 (13)	0.11356 (19)	0.09640 (17)	0.0570 (9)	
C7	−0.41344 (14)	0.0434 (3)	−0.13425 (18)	0.0807 (10)	
C8	−0.03201 (13)	−0.09808 (19)	0.32890 (17)	0.0529 (8)	
C9	0.01174 (15)	−0.19835 (19)	0.3795 (2)	0.0829 (10)	
C10	−0.01225 (12)	0.01440 (18)	0.35383 (16)	0.0470 (7)	
C11	−0.07411 (12)	0.07485 (18)	0.27784 (16)	0.0498 (8)	
C12	0.04790 (12)	0.06930 (19)	0.42923 (16)	0.0529 (8)	
C13	0.11229 (12)	0.01857 (19)	0.51147 (15)	0.0524 (8)	
C14	0.16670 (12)	0.07441 (19)	0.58368 (16)	0.0558 (8)	
C15	0.23504 (12)	0.03180 (18)	0.66822 (15)	0.0468 (8)	
C16	0.28189 (12)	0.10222 (17)	0.74142 (16)	0.0484 (7)	
C17	0.34695 (11)	0.06209 (17)	0.82033 (15)	0.0462 (7)	
C18	0.36860 (12)	−0.04585 (19)	0.83085 (17)	0.0533 (8)	
C19	0.32218 (13)	−0.11635 (19)	0.75823 (18)	0.0594 (9)	
C20	0.25664 (13)	−0.07833 (19)	0.67876 (17)	0.0558 (8)	
H2	−0.21290	−0.14941	0.09841	0.0664*	
H2A	0.00843	0.19892	0.38131	0.0985*	
H3	−0.32857	−0.13128	−0.04158	0.0726*	
H5	−0.31886	0.19129	−0.01079	0.0749*	
H6	−0.20295	0.17621	0.13074	0.0684*	
H7A	−0.41585	−0.01104	−0.17713	0.1210*	
H7B	−0.41647	0.11515	−0.15951	0.1210*	
H7C	−0.45667	0.03326	−0.12467	0.1210*	
H9A	0.01385	−0.20300	0.43799	0.1245*	
H9B	0.06423	−0.19504	0.39072	0.1245*	
H9C	−0.01457	−0.26184	0.34237	0.1245*	
H13	0.11552	−0.05758	0.51357	0.0629*	
H14	0.16074	0.15024	0.58025	0.0669*	
H16	0.26930	0.17631	0.73710	0.0581*	
H18	0.41302	−0.07088	0.88501	0.0640*	
H19	0.33541	−0.19024	0.76316	0.0712*	
H20	0.22594	−0.12729	0.63083	0.0669*	

### Atomic displacement parameters (Å^2^)

**Table:**

	*U*^11^	*U*^22^	*U*^33^	*U*^12^	*U*^13^	*U*^23^
O1	0.0611 (11)	0.0537 (9)	0.0678 (13)	0.0053 (8)	0.0077 (10)	0.0014 (9)
O2	0.0631 (12)	0.0662 (11)	0.0690 (15)	0.0031 (8)	0.0003 (10)	−0.0037 (10)
O3	0.0780 (12)	0.0638 (11)	0.0603 (13)	0.0089 (9)	0.0141 (10)	−0.0164 (10)
O4	0.0616 (12)	0.0917 (13)	0.0686 (14)	0.0114 (10)	−0.0097 (11)	−0.0236 (11)
N1	0.0395 (10)	0.0538 (10)	0.0368 (11)	0.0017 (8)	0.0119 (9)	0.0074 (9)
N2	0.0505 (11)	0.0524 (11)	0.0455 (13)	−0.0008 (9)	0.0091 (11)	0.0111 (10)
N3	0.0515 (12)	0.0620 (13)	0.0438 (14)	0.0006 (10)	0.0127 (11)	−0.0069 (11)
C1	0.0357 (11)	0.0600 (14)	0.0367 (14)	0.0024 (10)	0.0158 (11)	0.0056 (12)
C2	0.0492 (13)	0.0615 (14)	0.0434 (15)	0.0006 (11)	0.0156 (13)	0.0049 (13)
C3	0.0494 (14)	0.0755 (17)	0.0424 (16)	−0.0071 (12)	0.0138 (13)	−0.0026 (14)
C4	0.0393 (12)	0.0889 (18)	0.0383 (14)	0.0042 (13)	0.0147 (12)	0.0052 (15)
C5	0.0512 (14)	0.0725 (17)	0.0488 (17)	0.0145 (12)	0.0154 (14)	0.0123 (14)
C6	0.0497 (14)	0.0627 (15)	0.0462 (16)	0.0065 (11)	0.0161 (13)	0.0020 (12)
C7	0.0519 (14)	0.118 (2)	0.0482 (17)	0.0107 (15)	0.0090 (14)	0.0060 (17)
C8	0.0438 (13)	0.0600 (14)	0.0410 (15)	−0.0024 (10)	0.0121 (12)	0.0089 (12)
C9	0.0701 (17)	0.0590 (15)	0.071 (2)	−0.0015 (13)	0.0023 (16)	0.0208 (15)
C10	0.0364 (11)	0.0599 (14)	0.0382 (13)	−0.0017 (10)	0.0148 (11)	0.0039 (12)
C11	0.0426 (13)	0.0560 (14)	0.0432 (15)	0.0002 (11)	0.0169 (12)	0.0001 (12)
C12	0.0452 (13)	0.0583 (14)	0.0481 (16)	0.0013 (11)	0.0192 (13)	0.0009 (13)
C13	0.0395 (12)	0.0648 (14)	0.0394 (15)	−0.0024 (11)	0.0108 (12)	−0.0002 (13)
C14	0.0469 (13)	0.0640 (14)	0.0457 (16)	0.0008 (11)	0.0164 (13)	0.0018 (13)
C15	0.0394 (12)	0.0590 (14)	0.0367 (13)	−0.0045 (10)	0.0160 (11)	−0.0026 (12)
C16	0.0458 (12)	0.0510 (12)	0.0426 (14)	0.0001 (10)	0.0187 (12)	−0.0044 (12)
C17	0.0406 (12)	0.0556 (13)	0.0352 (13)	−0.0061 (10)	0.0146 (12)	−0.0081 (12)
C18	0.0424 (12)	0.0589 (14)	0.0462 (15)	0.0034 (11)	0.0141 (12)	0.0009 (13)
C19	0.0539 (14)	0.0516 (13)	0.0542 (17)	−0.0058 (11)	0.0150 (14)	−0.0048 (13)
C20	0.0475 (13)	0.0571 (14)	0.0496 (16)	−0.0116 (11)	0.0159 (13)	−0.0092 (13)

### Geometric parameters (Å, º)

**Table:**

O1—C11	1.259 (3)	C14—C15	1.458 (3)
O2—C12	1.310 (3)	C15—C20	1.393 (3)
O3—N3	1.209 (3)	C15—C16	1.387 (3)
O4—N3	1.219 (3)	C16—C17	1.373 (3)
O2—H2A	0.8200	C17—C18	1.367 (3)
N1—C1	1.421 (3)	C18—C19	1.380 (3)
N1—C11	1.360 (3)	C19—C20	1.373 (4)
N1—N2	1.401 (2)	C2—H2	0.9300
N2—C8	1.300 (3)	C3—H3	0.9300
N3—C17	1.462 (3)	C5—H5	0.9300
C1—C2	1.377 (3)	C6—H6	0.9300
C1—C6	1.381 (3)	C7—H7A	0.9600
C2—C3	1.377 (4)	C7—H7B	0.9600
C3—C4	1.375 (3)	C7—H7C	0.9600
C4—C5	1.375 (4)	C9—H9A	0.9600
C4—C7	1.508 (4)	C9—H9B	0.9600
C5—C6	1.386 (4)	C9—H9C	0.9600
C8—C10	1.428 (3)	C13—H13	0.9300
C8—C9	1.485 (4)	C14—H14	0.9300
C10—C11	1.433 (3)	C16—H16	0.9300
C10—C12	1.381 (3)	C18—H18	0.9300
C12—C13	1.446 (3)	C19—H19	0.9300
C13—C14	1.319 (3)	C20—H20	0.9300
			
C12—O2—H2A	109.00	C16—C17—C18	122.9 (2)
N2—N1—C11	110.88 (18)	N3—C17—C16	118.21 (19)
C1—N1—C11	130.70 (18)	C17—C18—C19	117.6 (2)
N2—N1—C1	118.39 (17)	C18—C19—C20	120.6 (2)
N1—N2—C8	107.02 (18)	C15—C20—C19	121.6 (2)
O3—N3—C17	119.0 (2)	C1—C2—H2	120.00
O4—N3—C17	118.1 (2)	C3—C2—H2	120.00
O3—N3—O4	122.9 (2)	C2—C3—H3	119.00
N1—C1—C6	121.2 (2)	C4—C3—H3	119.00
C2—C1—C6	119.0 (2)	C4—C5—H5	119.00
N1—C1—C2	119.8 (2)	C6—C5—H5	119.00
C1—C2—C3	120.2 (2)	C1—C6—H6	120.00
C2—C3—C4	122.3 (2)	C5—C6—H6	120.00
C3—C4—C7	121.8 (2)	C4—C7—H7A	109.00
C5—C4—C7	121.5 (2)	C4—C7—H7B	109.00
C3—C4—C5	116.7 (2)	C4—C7—H7C	109.00
C4—C5—C6	122.6 (2)	H7A—C7—H7B	109.00
C1—C6—C5	119.3 (2)	H7A—C7—H7C	109.00
N2—C8—C9	119.4 (2)	H7B—C7—H7C	109.00
N2—C8—C10	111.4 (2)	C8—C9—H9A	109.00
C9—C8—C10	129.3 (2)	C8—C9—H9B	109.00
C8—C10—C12	135.2 (2)	C8—C9—H9C	109.00
C11—C10—C12	120.0 (2)	H9A—C9—H9B	110.00
C8—C10—C11	104.8 (2)	H9A—C9—H9C	110.00
O1—C11—C10	127.3 (2)	H9B—C9—H9C	109.00
N1—C11—C10	105.93 (19)	C12—C13—H13	118.00
O1—C11—N1	126.7 (2)	C14—C13—H13	118.00
O2—C12—C13	115.6 (2)	C13—C14—H14	116.00
C10—C12—C13	125.7 (2)	C15—C14—H14	116.00
O2—C12—C10	118.8 (2)	C15—C16—H16	120.00
C12—C13—C14	123.6 (2)	C17—C16—H16	120.00
C13—C14—C15	127.9 (2)	C17—C18—H18	121.00
C14—C15—C20	122.5 (2)	C19—C18—H18	121.00
C16—C15—C20	117.6 (2)	C18—C19—H19	120.00
C14—C15—C16	119.9 (2)	C20—C19—H19	120.00
C15—C16—C17	119.7 (2)	C15—C20—H20	119.00
N3—C17—C18	118.9 (2)	C19—C20—H20	119.00
			
C1—N1—N2—C8	178.4 (2)	N2—C8—C10—C12	177.5 (3)
C11—N1—N2—C8	0.1 (3)	C9—C8—C10—C11	178.0 (3)
N2—N1—C1—C2	−3.6 (4)	C9—C8—C10—C12	−2.8 (5)
N2—N1—C1—C6	176.3 (2)	C8—C10—C11—O1	−178.1 (3)
C11—N1—C1—C2	174.2 (3)	C8—C10—C11—N1	1.7 (3)
C11—N1—C1—C6	−5.8 (4)	C12—C10—C11—O1	2.6 (4)
N2—N1—C11—O1	178.6 (3)	C12—C10—C11—N1	−177.7 (2)
N2—N1—C11—C10	−1.2 (3)	C8—C10—C12—O2	179.6 (3)
C1—N1—C11—O1	0.6 (5)	C8—C10—C12—C13	−1.2 (5)
C1—N1—C11—C10	−179.1 (3)	C11—C10—C12—O2	−1.3 (4)
N1—N2—C8—C9	−178.7 (2)	C11—C10—C12—C13	177.9 (3)
N1—N2—C8—C10	1.0 (3)	O2—C12—C13—C14	2.3 (4)
O3—N3—C17—C16	14.1 (4)	C10—C12—C13—C14	−177.0 (3)
O3—N3—C17—C18	−165.3 (2)	C12—C13—C14—C15	−177.9 (3)
O4—N3—C17—C16	−166.5 (2)	C13—C14—C15—C16	−173.4 (3)
O4—N3—C17—C18	14.1 (4)	C13—C14—C15—C20	7.5 (5)
N1—C1—C2—C3	179.2 (2)	C14—C15—C16—C17	−178.9 (2)
C6—C1—C2—C3	−0.8 (4)	C20—C15—C16—C17	0.2 (4)
N1—C1—C6—C5	−179.4 (3)	C14—C15—C20—C19	178.6 (3)
C2—C1—C6—C5	0.6 (4)	C16—C15—C20—C19	−0.5 (4)
C1—C2—C3—C4	0.6 (4)	C15—C16—C17—N3	−179.2 (2)
C2—C3—C4—C5	−0.1 (4)	C15—C16—C17—C18	0.1 (4)
C2—C3—C4—C7	−179.4 (3)	N3—C17—C18—C19	179.2 (2)
C3—C4—C5—C6	−0.2 (4)	C16—C17—C18—C19	−0.1 (4)
C7—C4—C5—C6	179.2 (3)	C17—C18—C19—C20	−0.2 (4)
C4—C5—C6—C1	−0.1 (4)	C18—C19—C20—C15	0.5 (4)
N2—C8—C10—C11	−1.7 (3)		

### Hydrogen-bond geometry (Å, º)

**Table:**

*D*—H···*A*	*D*—H	H···*A*	*D*···*A*	*D*—H···*A*
O2—H2*A*···O1	0.82	1.82	2.571 (3)	152
C6—H6···O1	0.93	2.33	2.958 (3)	125
C18—H18···O4^i^	0.93	2.57	3.496 (3)	171
C20—H20···O3^ii^	0.93	2.43	3.172 (3)	136

Symmetry codes: (i) −*x*+1, −*y*, −*z*+2; (ii) −*x*+1/2, *y*−1/2, −*z*+3/2.
